# Antifungal Activity of the Phenolic Compounds Ellagic Acid (EA) and Caffeic Acid Phenethyl Ester (CAPE) against Drug-Resistant *Candida auris*

**DOI:** 10.3390/jof7090763

**Published:** 2021-09-15

**Authors:** Fernanda Cristina Possamai Rossatto, Nagendran Tharmalingam, Iliana E. Escobar, Pedro Alves d’Azevedo, Karine Rigon Zimmer, Eleftherios Mylonakis

**Affiliations:** 1 Laboratory of Biofilms and Alternative Models, Federal University of Health Sciences of Porto Alegre, Porto Alegre 90050-170, RS, Brazil; fernandapr@ufcspa.edu.br (F.C.P.R.); pedro_dazevedo@yahoo.com.br (P.A.d.); karinerz@ufcspa.edu.br (K.R.Z.); 2 Division of Infectious Diseases, Rhode Island Hospital, Warren Alpert Medical School at Brown University, 593 Eddy Street, P.O. Box 328/330, Providence, RI 02903, USA; nagendran_tharmaligam@brown.edu (N.T.); iliana_escobar@brown.edu (I.E.E.)

**Keywords:** *Candida auris*, ellagic acid, CAPE, antifungals, antivirulence, biofilm

## Abstract

*Candida auris* is an emerging healthcare-associated fungal pathogen that has become a serious global health threat. Current treatment options are limited due to drug resistance. New therapeutic strategies are required to target this organism and its pathogenicity. Plant polyphenols are structurally diverse compounds that present a vast range of biological properties. In the present study, plant-derived molecules ellagic acid (EA) and caffeic acid phenethyl ester (CAPE) were investigated for their antifungal and antivirulence activities against *Candida auris*. We also tested against *C. albicans*. The minimum inhibitory concentration (MIC) for EA ranged from 0.125 to 0.25 µg/mL and for CAPE ranged from 1 to 64 µg/mL against drug-resistant *C. auris* strains. Killing kinetics determined that after 4 h treatment with CAPE, there was a complete reduction of viable *C. auris* cells compared to fluconazole. Both compounds might act by modifying the fungal cell wall. CAPE significantly reduced the biomass and the metabolic activity of *C. auris* biofilm and impaired *C. auris* adhesion to cultured human epithelial cells. Furthermore, both compounds prolonged the survival rate of *Galleria mellonella* infected by *C. auris* (*p* = 0.0088 for EA at 32 mg/kg and *p* = 0.0028 for CAPE at 4 mg/kg). In addition, EA at 4 μg/mL prolonged the survival of *C. albicans*-infected *Caenorhabditis elegans* (*p* < 0.0001). CAPE was not able to prolong the survival of *C. albicans*-infected *C. elegans.* These findings highlight the antifungal and antivirulence effects of EA and CAPE against *C. auris*, and warrant further investigation as novel antifungal agents against drug-resistant infections.

## 1. Introduction

Fungal infections are responsible for high mortality and morbidity, especially among immunocompromised patients, and their incidence has risen in the last few decades [1,2,3]. *Candida* spp. remain the most common cause of invasive fungal infections, and candidiasis is ranked as the fourth leading cause of bloodstream infections in the United States [2,4,5,6]. Among *Candida* spp., *Candida auris* has emerged as a highly drug-resistant pathogen in recent years, while *Candida albicans* is associated with more than 50% of human candidiasis [2,7]. *C. auris* was first described in 2009 as an isolate from the ear canal of a patient in Japan [8]. The first candidemia cases were described in South Korea, in 2011 [9]. Since then, invasive infections have increased, even outcompeting the most common fungal pathogen *C. albicans* at many centers in some countries [10]. Unlike *C. albicans*, this emerging pathogen can live on surfaces outside the human body, further complicating the management of these infections by healthcare facilities [11]. Furthermore, what makes *C. auris* uniquely challenging is a combination of the following: (i) widespread outbreaks in numerous hospitals worldwide due to its high transmissibility [11,12]; (ii) broad spectrum of clinical manifestations associated with a high mortality rate up to 70% [12,13]; (iii) ability to survive and proliferate for weeks either on dry or moist surfaces [14,15]; (iv) difficulty in correctly identifying *C. auris*, since this yeast can be misidentified as *Candida haemulonii* or *Candida parapsilosis* in laboratories that do not perform identification through molecular biology or MALDITOF techniques [14]; and (v) high rate of therapeutic failure and multidrug-resistance [16,17]. In fact, *C. auris* frequently present reduced susceptibility to azoles and echinocandin drugs [14]. Despite many studies addressing therapeutic approaches to treat *C. albicans* infections, few have attempted to investigate their antifungal activity against *C. auris* [7,12,17]. The success of *C. auris* as a pathogen results from their diverse virulence factors and fitness attributes, such as antifungal drug and environmental stress resistance, adherence and biofilm formation, and production of the extracellular hydrolytic enzymes proteinase and phospholipase [15,18]. However, the knowledge on *C. auris* virulence remains scarce, and further studies are required to investigate antivirulence strategies [19,20,21,22,23]. Considering that the development of new antifungal agents is restricted by the limited number of selective drug targets in yeast, there is an urgent need for novel antifungal candidates, including those targeting virulence factors [7,24,25,26,27].

Plants have traditionally served as a rich source for antimicrobial compounds due to secondary metabolites production. These metabolites are generally used as defense mechanisms against insects, herbivores, and microorganisms [28]. Among these compounds, polyphenols have protective properties on human health, mainly antioxidant, anti-inflammatory, anticancer, and antimicrobial [28,29]. Among these polyphenols, caffeic acid phenethyl ester (CAPE) and ellagic acid (EA) have been shown to have a wide range of biological activities. CAPE is one of the significant components of propolis. This compound presents immunomodulatory activity, inhibiting two key transcription factors involved in T-cell activation [30,31]. EA is a hydrolyzable tannin present in some plants such as pomegranates, nuts, and berries. The gut microbiota transforms EA to produce urolithins, bioavailable metabolites that can exert anti-inflammatory and anticarcinogenic effects [32]. These compounds have already demonstrated potential antifungal activity against *Candida* spp. [33,34,35,36,37], however, their mechanisms of action, synergism with antifungals, and in vivo studies still need to be investigated. Additionally, few studies report the effect of different plant-derived compounds on regulating processes involved in fungal virulence-related mechanisms, such as filamentation, biofilm formation, and production of extracellular enzymes [37,38,39,40,41,42,43]. In this respect, polyphenols have recently gained attention. After all, they display structural diversity and uniqueness in functional modes of action, rendering them promising options to counteract the emergence of *C. auris* drug resistance [44].

This work aims to investigate the antifungal activity of polyphenols from different classes against a more comprehensive panel of drug-resistant *C. auris* isolates. In addition, the study examined antivirulence activities, compound toxicity, a potential mechanism of action, and efficacy using the *Caenorhabditis elegans* and *Galleria mellonella* model hosts. According to our knowledge, the effect of polyphenols against *C. auris*, and its virulence factors, are for the first time elucidated here.

## 2. Materials and Methods

### 2.1. Chemicals

Caffeic acid (CA), caftaric acid (CF), ellagic acid (EA), epigallocatechin gallate (EGCG), engeletin (ENG), polydatin (also known as piceid or resveratrol-3-*O*-glucoside) (PD), amphotericin B (AMB), caspofungin (CPF), fluconazole (FLC) (Sigma-Aldrich Co., St. Louis, MO, USA), and caffeic acid phenethyl ester (CAPE) (Tocris (Bristol, UK) were used in this study and were dissolved in dimethyl sulfoxide (DMSO) or water at 10 mg/mL. RPMI 1640 (L-glutamine without sodium bicarbonate) (Sigma-Aldrich, MO, USA) at pH 7.0 with 0.165 M morpholinepropanesulfonic acid (MOPS; Sigma-Aldrich, MO, USA) was used as the test medium. 

### 2.2. Strains and Culture Conditions

The *Candida* strains used in this work were obtained from the American Type Culture Collection (ATCC, Manassas, VA, USA) and the Centers for Disease Control and Prevention Antibiotic Resistance Isolate Bank (CDC, Atlanta, GA, USA). They included a panel of 10 *C. auris* isolates (designated AR-BANK#0381 to AR-BANK#0390) (CAU-01 to CAU-10), as well as the wild-type strains *C. albicans* strain SC5314 (ATCC MYA-2876) (CAL), *Candida glabrata* ATCC 90030 (CG), *Candida krusei* ATCC 6258 (CK), *C. parapsilosis* ATCC 22019 (CP), and *Candida tropicalis* ATCC 13803, listed in Supplementary Table S1. Glycerol stocks were grown on yeast extract-peptone-dextrose agar (YPD, Research Products International, IL, USA) at 37 °C for 24 h.

### 2.3. Minimum Inhibitory Concentration

The broth microdilution assay was used to determine the minimum inhibitory concentration (MIC) of antifungals and polyphenols against the *Candida* strains according to the Clinical & Laboratory Standards Institute (CLSI) document M27-A2 [45] in 96-well flat-bottom microtiter plates (Corning no. 353072). Briefly, colonies of each strain were inoculated in 5 mL of YPD overnight at 37 °C in an orbital shaker at 225 rpm. The cells were harvested by centrifugation at 2700× *g* for 5 min, washed twice with sterile phosphate-buffered saline (PBS), and the final inoculum size was adjusted by hemocytometer to 1.0 × 10^3^ cells/mL in RPMI 1640. Antifungal agents and compounds were serially diluted in 50 μL, and 50 μL of cells were added. The concentration range tested was from 128 to 0.06 μg/mL for phenolic compounds. The plates were incubated at 37 °C for 24 h. Amphotericin B, caspofungin and fluconazole belong to the three major classes of antifungal agents (polyenes, echinocandins and azoles, respectively), and were used as positive controls. The concentration range tested was from 128 to 0.06 μg/mL for AMB and CPF, and 256 to 0.125 μg/mL for FLC. The least concentration of the compound that inhibited the visible growth was considered the MIC, and the most active compounds were selected and used for further studies. 

### 2.4. Time-Kill Curves

A time-to-kill kinetics assay was used to investigate the -cidal or -static nature of compound [46]. CAPE, EA, and fluconazole (control) were tested at various concentrations (0, 1, 2, 4, 8× MIC respectively). Log-phase cultures of *C. auris* CAU-04 or *C. albicans* SC5314 (10^6^ cells/mL) in RPMI 1640 with or without test compound were tested at 37 °C with agitation (225 rpm). At predetermined time points (0, 4, 8, 12, 24, 48 h), 50 μL aliquots were obtained from each solution, serially diluted in sterile water, and 5 μL were plated on YPD plates for 48 h at 37 °C for determination of colony-forming unit (CFU) counts. The fungicidal activity was defined as a ≥3-log_10_ (99.9%) reduction in numbers of CFU from the starting inoculum and fungistatic activity as a <99.9% reduction in growth from the starting inoculum [47].

### 2.5. Checkerboard Microdilution Assay

The interactions of EA and CAPE with amphotericin B, caspofungin, and fluconazole against isolates *C. auris* CAU-04 or *C. albicans* SC5314 were investigated by using the guidelines of CLSI reference technique [45], modified for a broth microdilution checkerboard procedure. The results obtained from the checkerboard assay were analyzed by the nonparametric approach of fractional inhibitory concentration (FICI), calculated according to the equation: ΣFIC = FICA + FICB, where FICA is the MIC of the combination/the MIC of drug A alone and FICB is the MIC of the combination/the MIC of drug B alone. The interpretation of FICI values was carried out as follows: synergy for FICI ≤ 0.5, indifference for FICI > 0.5 to 4.0, and antagonism for FICI ≥ 4.0 [48].

### 2.6. Mechanism of Action

#### 2.6.1. Sorbitol Protection Assay

The effect of EA and CAPE on the integrity of the fungal cell wall was evaluated by sorbitol protection assay. In brief, MIC values were determined after 2 and 7 days by the standard broth microdilution as described above in the absence and presence of 0.8 M sorbitol (Sigma-Aldrich, MO, USA) added to RPMI 1640. Caspofungin was used as a positive control. [49].

#### 2.6.2. Ergosterol Binding Assay

The ergosterol binding assay was conducted to assess if the compounds bind to the fungal membrane sterols. In brief, MIC values were determined after 48 h following the guidelines of CLSI as explained above, in the absence and presence of different concentrations of ergosterol (Sigma-Aldrich, MO, USA) added to the assay medium (0, 50, 100, 150, 200, and 250 μg/mL). Amphotericin B was used as a positive control. [49].

### 2.7. Virulence Factors

#### 2.7.1. Proteinase

The proteinase agar clearance assay was performed according to Yousuf et al. (2011) [50]. EA and CAPE were added to *C. auris* CAU-04 or *C. albicans* SC5314 inoculum in PBS (1 × 10^6^ cells/mL) at different concentrations. Aliquots of 2 μL were placed at equidistant points on proteinase agar plates and incubated at 37 °C for 3 days. Visible clear zones around the colonies indicated proteinase production by *Candida* strains. Proteinase activity (Pz value) was measured in terms of the ratio of the diameter of the colony to the diameter of the colony plus zone of precipitation. Values of Pz = 1 means that the test strain is negative for proteinase production.

#### 2.7.2. Phospholipase

The phospholipase assay was performed according to Yousuf et al. (2011) [50] using the egg yolk plates. EA and CAPE were added to *C. auris* CAU-04 or *C. albicans* SC5314 inoculum in PBS (1 × 10^6^ cells/mL) at different concentrations, and an aliquot of 2 μL was placed at equidistant points on phospholipase agar plates and incubated at 37 °C for 7 days. Visible precipitation zones around the colonies indicated phospholipase production by *Candida* strains. Phospholipase activity (Pz value) was measured as described above.

#### 2.7.3. Induction of Fungal Filamentation

The ability of hyphae formation by *C. auris* CAU-04 or *C. albicans* SC5314 was assessed by De Barros et al., (2018) [51]. In 24-well plates (Corning no. 3527, NY, USA), one milliliter of distilled water supplemented with 10% fetal bovine serum was mixed with 50 μL of brain heart infusion broth (BHI, Sigma-Aldrich, MO, USA) and 50 μL of either PBS (control group) or phenolic compound. Then, 100 μL of a *Candida* suspension (final concentration: 10^6^ cells/mL) were added and the plates were incubated for 24 h at 37 °C. Aliquots of 50 μL were transferred onto a glass slide and observed under a light microscope (40×). Ten microscopic fields per slide were chosen randomly to quantify the hyphae. The following score was used for the number of hyphae per microscopic field: score 0 (no hyphae), score 1 (1–3 hyphae), score 2 (4–10 hyphae), score 3 (11–20 hyphae), score 4 (more than 20 hyphae). 

#### 2.7.4. Biofilm Inhibition and Disruption

The ability of CAPE and EA to prevent fungal biofilm formation and eradicate a mature biofilm were investigated as previously reported with minor modifications [52,53]. Total biofilm biomass was measured using crystal violet (CV), while metabolic activity was measured by 2,3-bis-(2-methoxy-4-nitro-5-sulfophenyl)-2H-tetrazolium-5-carboxanilide (XTT) method. Overnight suspensions of *C. auris* CAU-04 or *C. albicans* SC5314 were centrifuged (4000× *g* for 5 min), washed twice with PBS, and adjusted to 2 × 10^6^ cells/mL in RPMI1640. Aliquots (50 μL) of fungal suspensions were transferred to each well of the microtiter plate. To determine the inhibition of biofilm formation, 50 μL of compounds or fluconazole were serially diluted, and each plate was incubated for 24 h at 37 °C. Following incubation, the biofilms were assessed using the CV and XTT methodologies. 

To assess the ability of compounds to disrupt a mature biofilm, the fungal inoculum was prepared as described above (adjusted to 1 × 10^6^ cells/mL) and aliquots (100 μL) were placed in each well. After 24 h of incubation at 37 °C, the cells were washed twice with PBS to remove planktonic yeast, and compounds or fluconazole were serially diluted. The plates were incubated for 24 h at 37 °C, and the biofilm was measured by CV and XTT methodologies. The minimum biofilm inhibitory concentration (MBIC_50_) and minimum biofilm eradication concentration (MBEC_50_) were defined as the minimum concentration leading to a 50% reduction of biofilm formation and 50% disruption of mature biofilm compared to the control sample, respectively.

##### Quantification of Biofilm Biomass

The biofilm biomass was quantified using the crystal violet assay with minor modifications [54]. Each well was washed twice with PBS to remove nonadherent cells, air-dried for 45 min, and stained with 100 μL of 0.4% aqueous crystal violet solution (Sigma-Aldrich, MO, USA) for 15 min. Any excess CV was removed by washing with sterile water before adding 100 μL of absolute ethanol to release the dye from the biofilm. After 30 min, the absorbance was measured at 570 nm (SpectraMax M2, Molecular Devices, CA, USA), and the biofilm inhibition was calculated.

##### Quantification of Metabolic Activity

The metabolic activity was quantified using a colorimetric assay based on the reduction of XTT, a tetrazolium salt, according to Khan et al. (2017) with minor modifications [54]. Following biofilm formation, the wells were washed twice with PBS. Afterward, 79 μL of PBS, 20 μL of XTT, and 1 μL of menadione were added to each prewashed well. The plates were incubated in the dark for 3 h at 37 °C. The absorbance was measured at 490 nm. 

##### Live/Dead Staining Assay

To detect the influence of CAPE on the vitality of *C. auris* CAU-04 or *C. albicans* SC5314 inside biofilms, live/dead staining was conducted. Briefly, after the washings with PBS, the biofilms were stained with live/dead staining solution (5 μM SYTO9 and 30 μM propidium iodide) and incubated for 30 min in the dark. The cells were rinsed with PBS and observed using a fluorescent microscope, and images were captured [55].

### 2.8. Hemolysis Assay

Hemolysis was determined as described by Tharmalingam et al. (2019) [55]. In a 96-well plate, 50 μL of polyphenols were serially diluted in PBS and added to 50 μL of 4% human erythrocytes suspended in PBS. Triton-X (0.002–1.0%) was used as reference sample. The plate was incubated at 37 °C for 1 h and then centrifuged at 500× *g* for 5 min. The supernatant from each well was transferred to a new plate, and the absorbance was read at 540 nm. 

### 2.9. Adherence Assay with A549 Cells

A549-lung cancer cell lines were used for the measurement of adhesion ability. The A549 cells were maintained in DMEM with 10% FBS and 1% penicillin/streptomycin. The cells were seeded in six-well plates, 24 h before infection. The MOI of 25 was used to infect the A549 cells. After 2 h of infection, test compounds (1×, 4× MIC) were added in a 5% CO_2_ incubator. After 22 h, the cells were washed and lysed with 0.02% of SDS in DMEM, and the CFU count was carried out.

### 2.10. Galleria mellonella Assays

#### 2.10.1. Toxicity

Killing assays using the alternative host *Galleria mellonella* were performed as previously described [56,57]. Sixteen randomly chosen *G. mellonella* (Vanderhorst Wholesale, OH, USA) with bodyweights of approximately 300 mg were used for each test group. We initially determined the sublethal concentrations of EA and CAPE. The compounds were inoculated into *G. mellonella* through the inferior left proleg at varying concentrations. Larvae were kept on petri dishes at 37 °C and monitored for survival over five days. Death was defined as a complete loss of mobility and lack of response to a physical stimulus using a plastic pipette tip.

#### 2.10.2. Infection Rescue Assay

We first injected different concentrations of each pathogen into the larvae to find the LD_100_ dose, and the curve was plotted. For the killing assay, larvae were inoculated with the drug on the inferior left proleg, followed by the lethal dose of the pathogen on the inferior right proleg. Different drug dosages were tested in the assay for each agent. After injections, larvae were incubated at 37 °C in plastic containers, and the number of dead larvae was scored daily over 5 days.

### 2.11. C. elegans-C. albicans Infection Assay

The *Caenorhabditis elegans* strain *glp-4;sek-1* (AU37) was maintained on *Escherichia coli* OP50 lawns that were spread on nematode growth medium agar plates and incubated at 15 °C. For the *C. elegans-C. albicans*. killing assay, worms were synchronized and grown to the young adult stage by incubating 24 h at 15 °C and 72 h at 25 °C. Worms were collected and washed three times with PBS in 50 mL tubes using gravity to pellet worms. Washed worms were then transferred to a *C. albicans* lawn grown on a BHI plate (grown overnight at 37 °C). Worms on *C. albicans*/BHI plate were incubated at 25 °C for 4 h and then washed 4 times with PBS. In a six-well plate, 30–75 worms were dispensed into each well containing desired drug concentration diluted in 20% BHI and 45 µg/mL kanamycin to a total volume of 2 mL. Worms were incubated at 25 °C and scored daily for dead worms for a total of 6 days. A worm was considered dead when it failed to respond to a gentle touch with a platinum wire picker.

### 2.12. Statistical Analysis

The Student’s *t*-test was used to compare the results from the time-kill and virulence factors assays. Kruskal–Wallis was used to compare the scores obtained in the filamentation assay. The Kaplan–Meier survival curves of *G. mellonella* and *C. elegans* were determined and the log-rank test evaluated the statistical significance. All tests were performed using GraphPad Prism 8 statistical software (GraphPad Software, Inc., San Diego, CA, USA) and a *p*-value of ≤0.05 was considered significant.

## 3. Results

### 3.1. CAPE and EA Show Antifungal Activity against C. auris

We initially evaluated the antifungal activity of seven polyphenols against a *Candida* spp. panel, including a number of *C. auris* isolates, which exhibited high-level resistance to fluconazole (Supplementary Table S2). Engeletin and polydatin did not show efficacy toward any of the *Candida* spp. (up to the highest tested concentration of 128 µg/mL), while caftaric acid only showed moderate effect toward CG and CAU-01 with a MIC of 16 µg/mL and 128 µg/mL, respectively (Table 1). To our knowledge, this is the first study to evaluate the antifungal activity of caftaric acid, engeletin, and polydatin against *Candida* spp. EA and CAPE showed the lowest MIC values of all compounds screened (Table 1). EA exhibited low MIC values against all *Candida* strains (MIC ≤ 0.5 µg/mL). MIC values of CAPE ranged from 1 to 64 µg/mL. Thus, we further pursued EA and CAPE as lead compounds. 

Growth inhibition was measured using checkboard assays to explore potential interactions between EA and CAPE with clinical antifungals used to treat *Candida* infections (amphotericin B, caspofungin, fluconazole). Combinations of either EA or CAPE and the antifungal agent on *Candida* strains, showed indifferent interactions.

### 3.2. CAPE Is Fungicidal and EA Is Fungistatic toward C. auris

**Figure 1:**
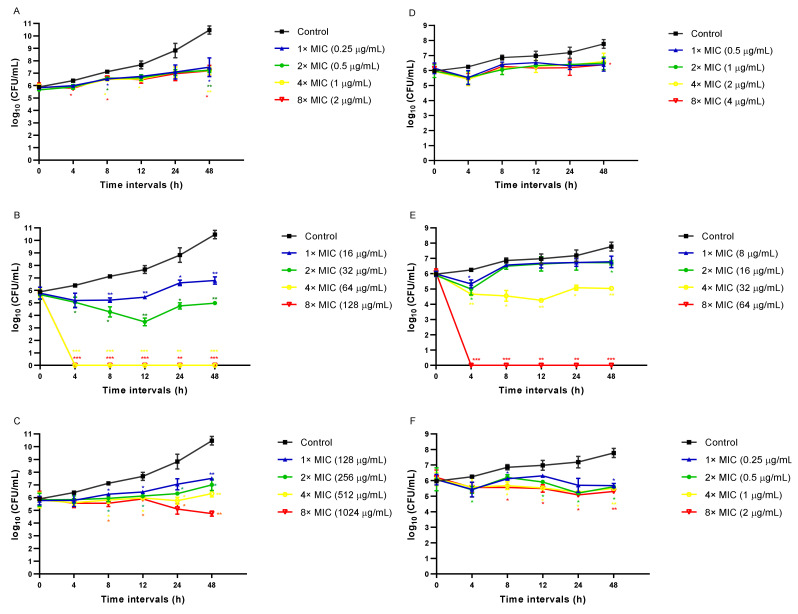
Time-kill curves obtained by incubating *C. auris* CAU-04 (**A**–**C**) and *C. albicans* SC5314 (**D**–**F**) strains with increasing concentrations of EA (**A**,**D**), CAPE (**B**,**E**) and FLC (**C**,**F**). Data represent the mean (± standard deviation, SD) of three independent experiments, and each one was carried out with triplicate determinations. For all experimental points, * *p* < 0.05, ** *p* < 0.01 or *** *p* < 0.001 were obtained for control versus treated samples.

The killing kinetics assay was conducted to address whether EA and CAPE exhibit fungicidal or fungistatic activity against *C. auris* or *C. albicans* (Figure 1). Both *C. auris* or *C. albicans* were inhibited by EA concentration used in the study (1× MIC–8× MIC) (Figure 1A,D), similar to the fungistatic effect observed with fluconazole (Figure 1C,F). Against *C. auris* CAU-04, CAPE exhibited fungicidal activity, rapidly reducing fungal viability below the detection limit within 4 h, at 4× and 8× MIC (64–128 µg/mL) (Figure 1B). The same effect occurred in *C. albicans* cells when treated with CAPE at 8× MIC (64 µg/mL) (Figure 1E). 

### 3.3. EA and CAPE May Affect Fungal Cell Wall

Sorbitol is an osmotic protectant used for stabilizing fungal protoplasts, thereby providing a suitable environment for the cell wall biosynthesis pathway [58]. For this reason, an increase in MIC values is expected when a mechanism of action (MOA) is through cell wall damage [58]. The sorbitol protection assay was performed to test the effect of compounds on the fungal cell wall integrity. When *C. auris* or *C. albicans* were treated with EA or CAPE in a medium supplemented with sorbitol, MIC increased after seven days of incubation compared to MIC in a medium without sorbitol (Table 2). The MIC values of EA and CAPE increased 128-fold and twofold for both *C. auris* or *C. albicans*, respectively. MIC values of caspofungin (positive control) increased fourfold and 32-fold against *C. auris* CAU-04 or *C. albicans* SC5314 respectively.

The ability of the tested compounds to bind to ergosterol was also investigated through ergosterol binding assay. No variations in MIC were observed for compounds in this assay, suggesting that EA and CAPE would not act by binding to fungal ergosterol (Table 3). As expected, a 4–128-fold increase of MIC values was observed for amphotericin B (positive control).

### 3.4. Effect of EA and CAPE on Proteinase and Phospholipase Production

**Figure 2:**
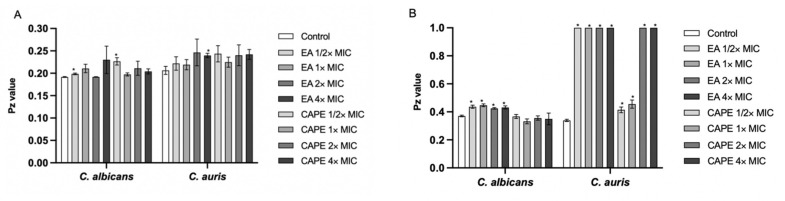
Effect of EA and CAPE on proteinase and phospholipase production. (**A**) Effect on proteinase secretion by *C. auris* CAU-04 *and C. albicans* SC5314 when exposed to different EA and CAPE concentrations. (**B**) Effect on phospholipase secretion by *C. auris* CAU-04 and *C. albicans* SC5314 when exposed to different concentrations of EA and CAPE. * *p* < 0.05.

Figure 2 summarizes the proteinase and phospholipase secretion results by *C. auris* CAU-04 and *C. albicans* SC5314 following exposure to different EA and CAPE concentrations. Our results show that both compounds could not inhibit *Candida* proteinase production at all concentrations tested (Figure 2A). EA significantly reduced the phospholipase production in *C. auris*, since there was no zone of precipitation around the fungal colonies. *C. auris* exposed to at least 32 µg/mL CAPE (2× MIC) had decreased phospholipase secretions. No significant phospholipase inhibition was observed after either EA or CAPE exposure in *C. albicans* (Figure 2B). 

### 3.5. CAPE Inhibits Hyphal Development in C. albicans

A hyphae-inducing RPMI medium was used to examine hyphal inhibition by EA and CAPE. In the filamentation assay, *C. auris* was unable to produce hyphae under test conditions but we observed many *C. albicans* hyphae in the control groups with PBS after 24 h (Supplementary Figure S1A). CAPE demonstrated excellent ability to reduce hyphae production at all concentrations. When *C. albicans* was cultured with CAPE at 1× MIC (8 µg/mL), a hyphae reduction was found (*p* = 0.0238). CAPE at 2× MIC (16 µg/mL) completely suppressed yeast-to-hyphal conversion (*p* < 0.0079). However, EA treatment did not cause any statistically significant reduction in hyphae formation (Supplementary Figure S1A). Furthermore, we confirmed that this activity was not due to the direct killing of *C. albicans* by EA and CAPE by measuring CFU/mL (Supplementary Figure S1B). The microscopic analysis of filamentation demonstrated that the EA at 2 µg/mL led to a discrete reduction in the quantity of hyphae formation. When *C. albicans* was cultured with CAPE at 16 µg/mL, fungal cells grew by budding, whereas control large hyphal cells were observed (Supplementary Figure S1C). 

### 3.6. Effect of EA and CAPE on C. auris and C. albicans Biofilms

**Figure 3:**
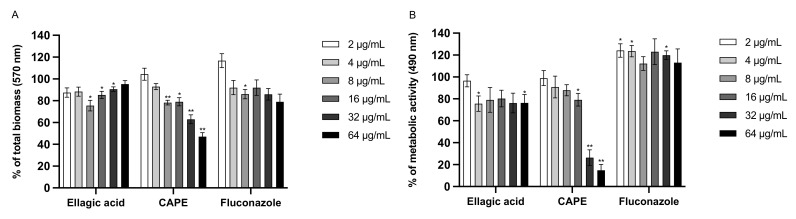

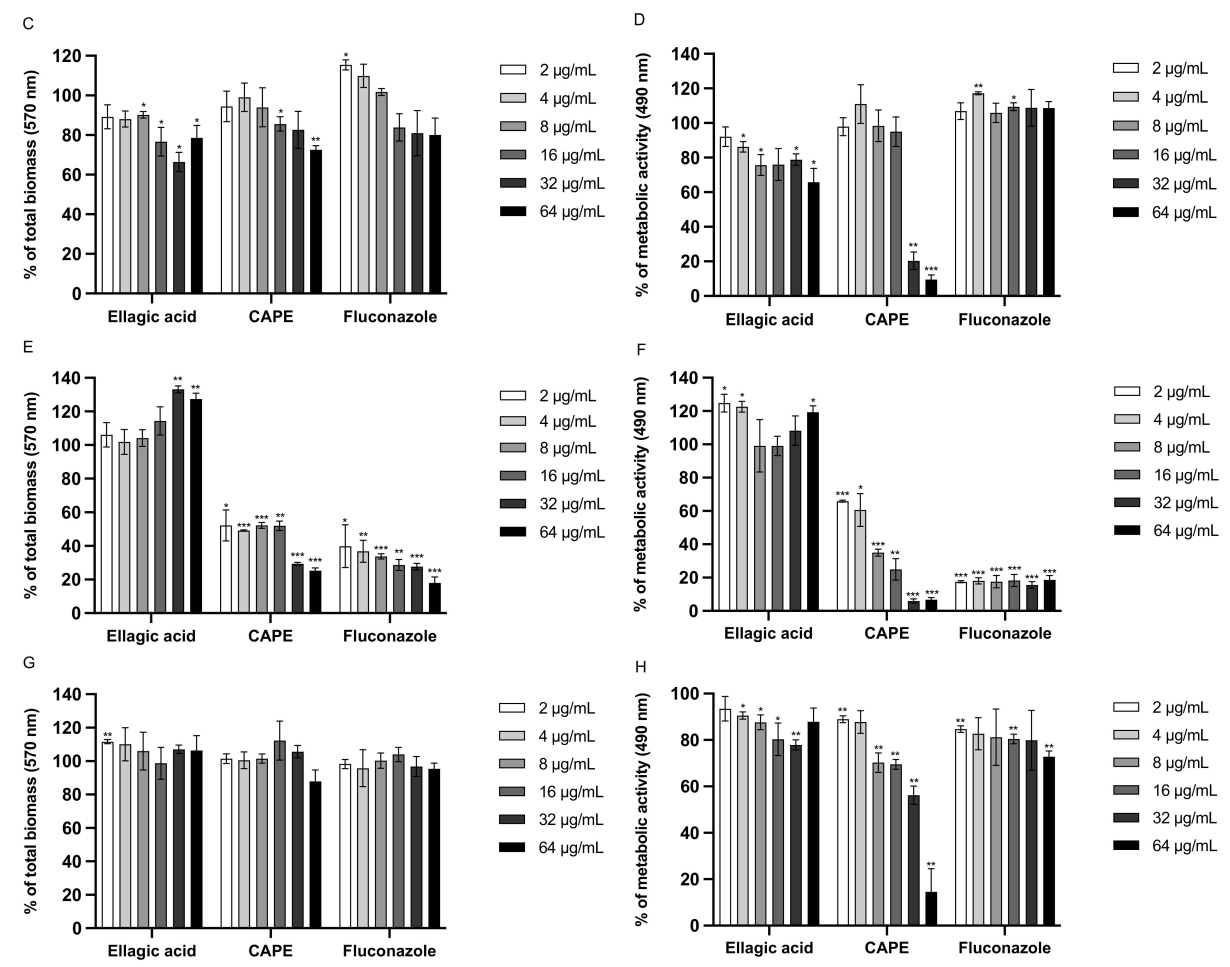
Inhibition of biofilm formation (**A**,**B**,**E**,**F**) and eradication of mature biofilm (**C**,**D**,**G**,**H**) by EA, CAPE, and FLC against *C. auris* CAU-04 (**A**–**D**) and *C. albicans* SC5314 (**E**–**H**) strains. Two different methodologies were used to quantify biofilm: crystal violet assay (**A**,**C**,**E**,**G**), which measures biofilm total biomass, and XTT assay (**B**,**D**,**F**,**H**), which measures metabolic activity. Data represent the mean (± standard deviation, SD) of three independent experiments, and each one was carried out with triplicate determinations. For all experimental points, * *p* < 0.05, ** *p* < 0.01 or *** *p* < 0.001 were obtained for treated samples versus control (100% of biofilm formation and metabolic activity).

We investigated whether EA and CAPE could prevent biofilm production and disrupt preformed *Candida* biofilms (Figure 3). The ability of EA and CAPE to inhibit biofilm formation was determined (Figure 3A,B,E,F). Regarding the inhibition of biofilm formation assay using CV, CAPE exhibited a dose-dependent inhibition of both *C. auris* and *C. albicans* biofilms. When tested against *C. auris*, CAPE was effective at 64 µg/mL (4× MIC), reducing biofilm by 53% (*p* = 0.0023), while fluconazole, at the same concentration, reduced biofilm formation by 21% (Figure 3A). Interestingly, CAPE was able to reduce the biofilm formation at subinhibitory levels. CAPE at ¼ × MIC (2 µg/mL) reduced biofilm formation by 48% against *C. albicans,* relative to the untreated control (*p* = 0.0178) (Figure 3E). EA was ineffective at all concentrations tested. The metabolic activity of *Candida* was measured using the XTT reduction assay. Consistent with the CV results, CAPE exhibited an inhibitory effect on biofilm formation by *C. auris* and *C. albicans,* reducing the metabolic activity of sessile cells in a dose-dependent manner (Figure 3B,F). CAPE at 64 µg/mL reduced metabolic activity of *C. auris* by 85% (*p* = 0.0019) while fluconazole, at the same concentration, was ineffective (Figure 3B). The ability of EA and CAPE to disrupt mature biofilm was also measured (Figure 3C,D,G,H). CAPE reduced metabolic activity by 79.6% (at 32 µg/mL) and 90.5% (at 64 µg/mL) (*p* < 0.001) against *C. auris*, relative to the untreated control (Figure 3D). CAPE was effective at reducing metabolic activity only at high concentrations, where the compound at 64 µg/mL reduced metabolic activity of *C. albicans* by 85.4% (*p* = 0.007) (Figure 3H). In contrast, the total biomass was not reduced by CAPE (Figure 3C,G). Fluconazole was used as a control and did not cause any disruption in *C. auris* or *C. albicans* mature biofilms at all tested concentrations.

The minimum concentrations leading to a 50% reduction of biofilm formation and 50% disruption of mature biofilm, MBIC_50,_ and MBEC_50_, respectively, were determined by quantifying the total biomass and the metabolic activity, using the CV and the XTT assay (Supplementary Table S3). Consistent with its antifungal activity against planktonic cells, CAPE exhibited an inhibitory effect on biofilm formation of both *C. auris* or *C. albicans* with MBIC_50_ values of 32 μg/mL or 8 μg/mL against *C. auris* or *C. albicans,* respectively, in the XTT assay; and values of 64 μg/mL or 32 μg/mL using the CV assay. CAPE also showed activity against mature biofilms with MBEC_50_ values of 32 μg/mL or 64 μg/mL against *C. auris* or *C. albicans*, respectively, in the XTT assay. The MBEC_50_ was higher for both strains (>64 μg/mL) in the CV assay. On the other hand, EA did not demonstrate antibiofilm activity against *C. auris or C. albicans*, with an MBIC_50_ and MBEC_50_ higher than 64 μg/mL for both methodologies. 

**Figure 4:**
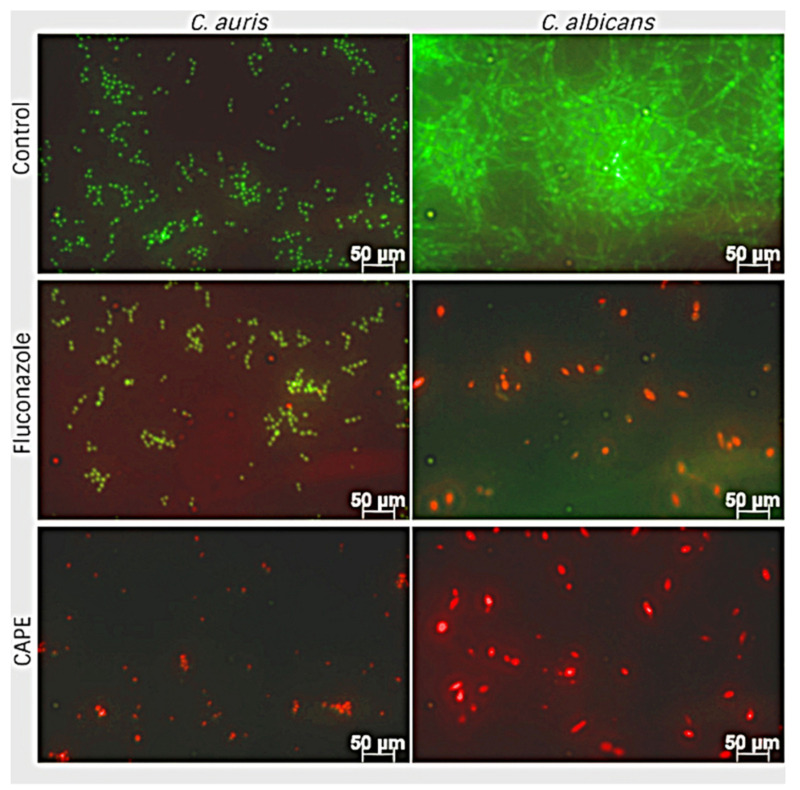
Fluorescence microscopy comparison of *C. auris* and *C. albicans* biofilms grown in the presence of CAPE and fluconazole. Images taken in two different channels (red and green) were overlapped. *C. auris* and *C. albicans* biofilms were treated with CAPE 2× MIC (32 μg/mL and 16 μg/mL, respectively) and fluconazole (32 μg/mL and 16 μg/mL, respectively).

Since CAPE presented antibiofilm activity, we performed live/dead staining of cells after biofilm formation to differentiate viable versus dead cells, according to manufacturer instructions (Thermo Fisher Scientific, Waltham, MA, USA). The kit contains two fluorescent probes, SYTO9, and propidium iodide (PI). The live cells absorbed dye STYO9, therefore emitting green light, while dead cells absorb PI, thus emitting red light. To evaluate the viability of yeast cells after treatment with CAPE, fluorescence microscopy images were acquired after 24 h of biofilm formation. As shown in Figure 4, the *C. auris* biofilm was predominately composed of budding yeasts and occasional pseudohyphae. In contrast, the biofilm of *C. albicans* consisted of a network of hyphae and budding yeasts cells connected at several points. DMSO-treated fungal cells (control) were stained green, while CAPE-pretreated fungal cells (2× MIC) were stained red, suggesting that pretreatment with CAPE killed those cells attached to the surface. CAPE also inhibited the yeast-to-hyphae conversion, corroborating the filamentation results (Supplementary Figure S1). In addition, CAPE decreased biofilm formation since the density of cells attached was lower than the control for both strains. Our data indicate that CAPE inhibited biofilm formation by reducing the rate of its development. When *C. auris* was treated with fluconazole, most cells were alive, supporting with XTT assay results (Figure 3).

### 3.7. Toxicity of EA and CAPE on Human Red Blood Cells (hRBC)

**Figure 5:**
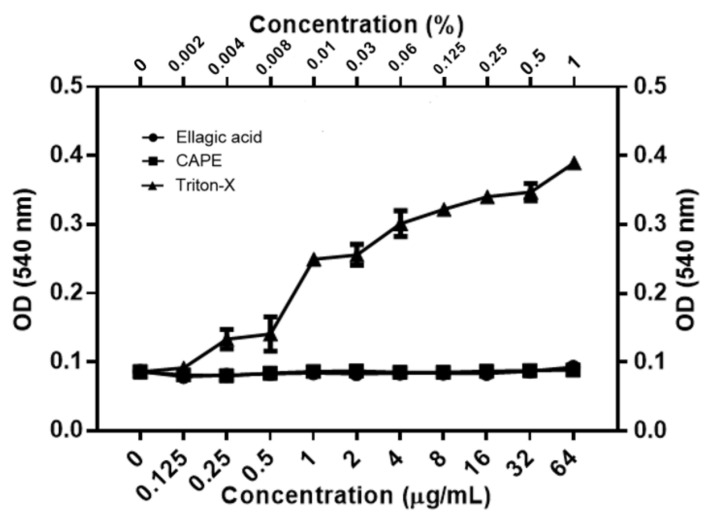
Cytotoxicity of EA and CAPE in human red blood cells. Triton-X was used as a positive control. Data represent the mean ± SD (*n* = 3).

Mammalian erythrocytes are an excellent model to evaluate the cytotoxicity of molecules by measuring cellular damage [59,60]. Hemolysis assay disclosed that all tested concentrations of EA and CAPE did not cause any damage to the human erythrocyte membrane. We used Triton-X as a positive hemolytic agent (Figure 5). 

### 3.8. CAPE Inhibits Adhesion of C. auris and C. albicans on A549 Cells

**Figure 6:**
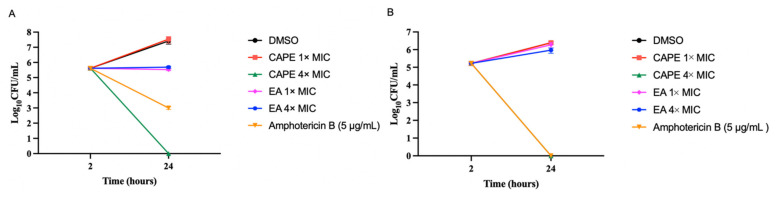
Effects of EA and CAPE on *C. auris* (**A**) and *C. albicans* (**B**) virulence using the cell line A549. The results are reported in log CFU/mL. The compounds were dissolved in DMSO, and the amount of DMSO used as the solvent for the compounds was used as a control. Amphotericin B was used as a positive control.

The ability to adhere to epithelial cells contributes to the first step in *Candida* spp. colonization or infection [61]. For this reason, we also tested how the candidate compounds would affect both *C. auris* or *C. albicans* adherence to human lung epithelial A549 cells by measuring the total viable count. After 22 h of infection, the A549 cells were washed, and the fungal-adhered cells were enumerated. At 1× MIC, both compounds did not potentially inhibit fungal adhesion (Figure 6). However, at 4× MIC concentration, CAPE potentially inhibited *C. auris* and *C. albicans* adherence as demonstrated by the total viable count. (Figure 6). 

### 3.9. EA and CAPE Protect Galleria mellonella Larvae from Candida spp. Infection

First, we evaluated the acute systemic toxicity of compounds in *G. mellonella*. The larvae were injected with varying EA and CAPE concentrations, and their survival was monitored for 5 days. Both compounds did not exert toxic effects on the larvae when administered at those concentrations, up to 128 mg/kg (Supplementary Figure S2A). These results corroborate with our previous in vitro data where we demonstrated that EA and CAPE were not toxic towards human red blood cells (Figure 5). *G. mellonella* showed a different survival profile depending on the fungal strains they were infected with (Supplementary Figure S2B,C).

**Figure 7:**
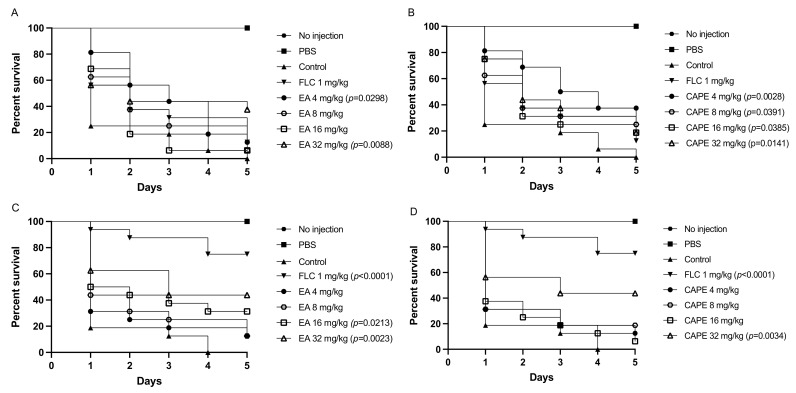
Kaplan–Meier survival curves of infected *G. mellonella* larva. (**A**,**B**) Survival of *G. mellonella* larvae infected with *C. auris* and treated with different doses of EA or CAPE, respectively, 2 h prior the infection. (**C**,**D**) Survival of *G. mellonella* larvae infected with *C. albicans* and treated with different doses of EA or CAPE, respectively, 2 h prior the infection.

Different concentrations of EA and CAPE were injected into the larvae at 2 h before infection. In the control group, infection with *C. auris* or *C. albicans* caused death in 100% of the larvae within 5 days (Figure 7). After infection with *C. auris*, the groups of larvae pretreated with EA at 32 mg/kg demonstrated an increase in survival of 44% when compared to the control group (*p* = 0.0088) (Figure 7A). Surprisingly, the lowest concentration of CAPE tested (4 mg/kg) led to the highest increase in *G. mellonella* survival after infection with *C. auris*, with a larvae survival of 37.5% (*p* = 0.0028) (Figure 7B). This may be explained by solubility problems of CAPE at high concentrations or fungal stress responses during host infection after pretreatment with high concentrations of CAPE. When the larvae were pretreated with either EA or CAPE (32 mg/kg) before *C. albicans* infections, the survival rate of *G. mellonella* larvae was significantly prolonged, with an approximate larvae survival of 44% for both compounds (*p* = 0.0023 for EA and *p* = 0.0034 for CAPE) (Figure 7C,D). Fluconazole at 1 mg/kg protected *G. mellonella* from *C. albicans* infection (*p* < 0.0001) (Figure 7C,D). However, it was not effective against the fluconazole-resistant *C. auris* strain, resulting in 87.5% death after five days (Figure 7A,B).

### 3.10. EA Protects C. elegans from C. albicans Infection

**Figure 8:**
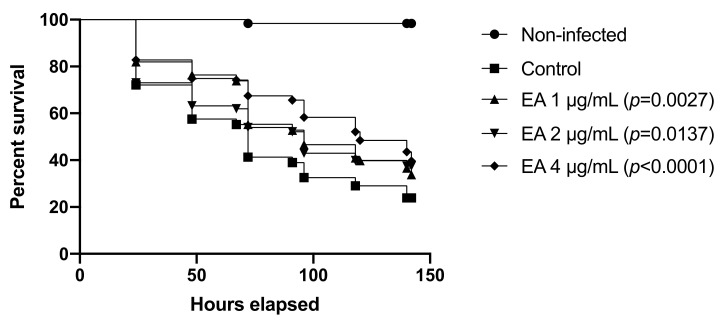
Kaplan–Meier survival curves of infected *C. elegans*. EA protects *C. elegans* larvae from *C. albicans* infection after 150 h.

Finally, to further evaluate EA and CAPE as potential anti-infective compounds against *Candida* spp., we tested both compounds in a second invertebrate model, *C. elegans*. The infection of *C. elegans* by *C. albicans* resulted in a reduction of worm viability. At six days postinfection, the survival ratio of this group was 24%, while the uninfected animals showed 98% survival on this day (Figure 8). However, when the animals were treated with 4 μg/mL EA, the survival of worms was approximately 40% (*p* < 0.0001). Surprisingly, CAPE up to 128 μg/mL did not significantly increase the viability of *C. elegans* compared with the control group. 

## 4. Discussion

Candidiasis is a significant healthcare problem worldwide. The limited number of antifungal drugs combined with the development of resistance to these agents presents a challenge to treat *Candida* spp. infections [7,13]. Among *Candida* spp., *C. auris* has emerged as a global health threat. It causes highly invasive infections with high mortality rates in hospitalized patients, especially those with comorbidities and immunosuppression [13,14,15]. A high percentage of *C. auris* isolates have shown resistance to one or more of the three major antifungal drugs (azoles, echinocandins, polyenes), making the treatment challenging [14]. Thus, novel therapeutic approaches are needed to treat *C. auris* infections, including the use of naturally-occurring agents. Medicinal plants are rich in diverse chemical structures, which warrants more thorough investigation as potential novel antifungal agents [27]. Polyphenols are naturally occurring secondary plant metabolites and are generally involved in defense against ultraviolet radiation or aggression by pathogens [29].

This study assessed the activity of seven plant derived polyphenols against *C. auris* mainly and *C. albicans*. The results demonstrated that polyphenol ellagic acid and caffeic acid phenethyl ester are more effective and present antifungal against *C. auris* and *C. albicans*. The effect of these compounds on the virulence attributes were also investigated. CAPE presented anti-adhesives properties and inhibited fungal filamentation. Besides having low MIC, at subinhibitory concentration, ellagic acid inhibited phospholipase production by *C. auris*. Both compounds were not toxic to human erythrocytes and the invertebrate larva *G. mellonella.* In addition, both compounds were able to prolong the larvae survival after infection with *C. auris* or *C. albicans*. EA was also able to protect *C. elegans* against *C. albicans* infection.

The MIC results of this study demonstrated the antifungal activity of EA and CAPE against *Candida* spp., including a panel of 10 *C. auris* isolates. To our knowledge, this is the first study to evaluate the antifungal activity of EA and CAPE against *C. auris.* Both compounds were active against *C. auris* fluconazole-resistant strains, including those also resistant to caspofungin or amphotericin B. *C. auris* CAU-04 displays resistance to two different classes antifungals: azoles (fluconazole) and echinocandins (caspofungin). It is noteworthy that an echinocandin drug is recommended initial therapy to treat *C. auris* infection [62]. Brighenti et al. (2017) showed that EA is effective against *C. albicans* ATCC 18804, with a MIC value of 3.2 µg/mL [63], supporting our observations. CAPE was also effective against *C. albicans,* presenting MIC values ranging 32–64 µg/mL, similar to our findings [30,64].

Interestingly, some studies report that the high quantity of polyphenols present in all plant parts can explain most of the antifungal effects of diverse plant extracts. Several of these polyphenols are identified as ellagic acid and caffeic acid derivatives [65,66,67,68,69,70,71]. Rangkadilok et al. (2012) showed that ellagic acid displayed the most potent antifungal activity among polyphenols detected in longan seed extracts [72]. Gatto et al. (2020) reported that the ethyl acetate fraction of stems of *Myrcia hatschbachii* inhibited the growth of *C. albicans* at 15.6 µg/mL, displaying fungistatic action. This fraction presented high quantity of phenolic compounds in its composition, considering that the main compounds identified by nuclear magnetic resonance were ellagic and 3-*O*-methyl ellagic acids [73]. Freires et al. (2016) demonstrated that the antifungal potential of propolis extracts may be related to the presence of flavonoids such as CAPE in their chemical composition, suggesting that the mechanism of action is related to disruption of fungal cell wall [65]. The anti-*Candida* activity of natural products that have phenolic acids in their compositions is probably due to the precipitation of proteins and the antioxidant capacity of these compounds which renders modifications in the metabolism of the pathogens [74]. 

Ellagic acid and CAPE present low solubility in water and limited permeability, which can interfere their bioavailability [75]. For this reason, pharmaceutical delivery of these molecules can be improved to overcome these problems. Some studies have proposed different carriers for these compounds without interfering with their inhibitory effect against *Candida* spp. [76,77,78]. Sampaio et al. (2021) evaluated ellagic acid-cyclodextrin complexes for antifungal activity. They found that the MIC of the complex was 25 µg/mL against *C. albicans* 18804 and 48 h-biofilms were inhibited at 250 µg/mL [76]. Saelo et al. (2016) investigated the MIC of CAPE nanoparticles (NP) against *C. albicans* TISTR 5779 and found that CAPE-NP might kill pathogens better than CAPE, with a MIC value of 700 µg/mL, while CAPE alone presented a MIC of 1400 µg/mL [77]. Furthermore, structural analogs can be synthesized to increase their efficacy [79]. De Vita et al. (2016) designed a new series of cinnamoyl ester and amide derivatives preserving the cinnamoyl moiety and removing the hydroxyl groups of caffeic acid to obtain an improved chemical stability. The most active compound showed more than 50% biofilm inhibition at 2 µg/mL against *C. albicans* ATCC 10231 [79].

We also tested the synergistic effect between EA and CAPE with antifungals through checkboard assays. However, all combinations evidenced indifferent interactions. These results are in contrast with the previous findings by Sun et al. (2018), where CAPE synergistically enhanced the antifungal activity of fluconazole against resistant *C. albicans* [80]. Furthermore, the synergistic effect between CAPE and caspofungin against *C. albicans* through disrupting iron homeostasis has already been described [80]. These differences can be related to different growth conditions, purity of the compounds, and origin of the strains.

Based on the MIC values of EA and CAPE in media with and without sorbitol, our findings indicate that these compounds might affect the fungal cell wall. A similar study was performed by Silva Junior et al. (2010), which found that the MIC of EA against *Saccharomyces cerevisiae* was raised from 62 to 250 µg/mL (fourfold) in the presence of sorbitol 0.8 M, suggesting that EA may act by modifying the fungal cell wall [58]. A possible effect on the *C. albicans* cell wall has been shown for caffeic acid derivatives, which may interfere with 1,3-β-glucan synthase, an enzyme unique to fungi [81]. While the mode of action of caffeic acid derivatives has been previously described, our study is the first to gain insight into the mechanism of action of caffeic acid phenethyl ester and ellagic acid on both *C. auris* and *C. albicans*. However, further investigations are needed to understand their specific role on fungal cell walls.

Virulence of *C. auris* and *C. albicans* depends on their ability to secrete tissue-damaging hydrolytic enzymes, adhere to host tissues, and change its morphological form from budding yeast to hyphal filaments as in the case of *C. albicans* [50]. For these reasons, we investigated whether EA and CAPE might present antivirulence properties, including inhibition of enzymes secretion, filamentation, and biofilm formation. There are two prominent families of hydrolytic enzymes produced by *Candida* spp.: the secretory aspartyl proteinases and phospholipases. These enzymes degrade physiological substrates such as immunoglobulins and albumin, damage host cell membranes, and are involved in counteracting the host immune response by blocking phagocytosis and intracellular killing [50]. EA and CAPE did not inhibit proteinase production by both *C. auris* or *C. albicans* however, these polyphenols were able to reduce phospholipase production by *C. auris*. *C. auris* cells exposed to sublethal concentration of EA (½ × MIC) were found to have no secretion of phospholipase.

The ability to form hyphae is a crucial step in *Candida* pathogenesis, since hyphae are involved in adherence, early stages of invasion and penetration into the host tissues [41]. *C. auris* could not produce hyphae under in vitro conditions used in this study; however, *C. albicans* SC5314 produced numerous hyphae. Some environmental factors, such as serum, are potent inducers of *C. albicans* filamentation [51]. In our study, we used serum to induce filamentation, however, it was recently determined that this factor does not induce filamentous growth in *C. auris* [82]. *C. auris* has the potential to undergo filamentation under certain unidentified conditions, but environmental conditions and genetic triggers for filament formation remain elusive [82,83] and the switch between typical yeast, filamentation-competent yeast and filamentous cells may be temperature-dependent and facilitated by passage through a mammalian body [82]. As we learn more about the filamentation in *C. auris*, different growth conditions can be tested to further stimulate *C. auris* filamentation, and thus evaluate the inhibitory effect of EA and CAPE on *C. auris* filamentation. Since passage through mouse or *G. mellonella* induces the filamentous morphology [82,84], a whole animal model can be used for the study of disseminated *C. auris* candidiasis after understanding the pharmacokinetics and pharmacodynamic of EA and CAPE. Our data revealed that CAPE significantly inhibited the transition of cells from budding yeast to hyphae in *C. albicans*, thus preventing one of the initial stages of infection. CAPE at 2× MIC (16 µg/mL) completely suppressed yeast-to-hyphal conversion without interfering with fungal growth. Microscopic images confirmed that CAPE inhibited hyphae formation, in agreement with other studies [85]. 

*Candida* spp. form biofilms, which protect them from external factors such as host immune system defenses and antifungal drugs. For this reason, resistance to antifungals is a hallmark phenotype of biofilms. Indeed, *Candida* biofilms present on medical devices often are recalcitrant to treatment with antifungals [19,23]. *Candida* spp. survive in several anatomically different sites, switch between yeast and hypha forms in response to environmental changes, and gradually form biofilm. Indeed, adhesion to biotic or abiotic surfaces is the inceptive phase of biofilm formation [86]. It is usually necessary to use higher concentrations of compounds than their MIC to be effective against *Candida* biofilms, since sessile cells are more resistant to antimicrobial agents than planktonic cells [76]. Besides its antifungal activity, we found that CAPE also presented antiadhesive properties against both *C. auris* and *C. albicans*. CAPE at 4× MIC reduced biofilm by 53% against *C. auris*, and, at ¼ × MIC, reduced biofilm formation by 48% against *C. albicans* and fluconazole, at the same concentration, reduced biofilm by 21%. This result was expected since the *C. auris* strain employed showed resistance to fluconazole in MIC assays. Fluconazole is one of the first-line treatment options in several cases of invasive candidiasis [27]. In agreement with the biofilm results, CAPE impaired *C. auris* and *C. albicans* adhesion to cultured human epithelial cells at 4× MIC. Similar results were found by De Barros et al. (2021), which reported a reduction of 68.5% in the amount of total biomass of *C. albicans* biofilm when treated with CAPE at 5× MIC (80 µg/mL) [85]. De Vita et al. (2014) reported that caffeic acid derivatives could inhibit *C. albicans* ATCC 10231 biofilms. The synthesized esters 7, 8, and 10 presented MBIC_50_ ranging from 16 to 32 μg/mL and MBEC_50_ ranging from 64 to 128 μg/mL at 24 h [37]. Breger et al. (2007) showed that CAPE strongly inhibited both *C. albicans* filamentation and biofilm formation. At 30 μg/mL, CAPE inhibited *C. albicans* DAY185 biofilm formation, reducing biofilm biomass compared to the control [21]. CAPE also inhibited the formation of *Streptococcus mutans* biofilm and its metabolic activity in mature biofilms [87]. For this reason, the antibiofilm activity of CAPE against both yeast and bacteria indicates its broad-spectrum antimicrobial potential.

Both compounds were not toxic to human erythrocytes at all concentrations tested. These findings agree with earlier studies that indicate the absence of toxicity of these compounds in RBC and demonstrate protective roles of phenolic compounds in damaged cells [88,89,90]. Based on these promising data, we interrogated EA and CAPE using two different in vivo invertebrate models: the wax moth larva *G. mellonella* and the nematode *C. elegans. G. mellonella* provides a facile model further to evaluate the toxicity and efficacy of antifungal agents, as it is a quick and economical experimental host to be used before more expensive mammalian models [91,92]. EA and CAPE, at all concentrations tested, were not toxic to the host. When the efficacy of both compounds was tested on candidiasis, both EA and CAPE were able to prolong larvae survival. When infected with *C. auris*, treatment with CAPE at 4 mg/kg increased the survival rate by 37.5%, 5 days postinfection. Next, we used the *C. elegans* model to evaluate the effects of EA and CAPE on *C. elegans* infected with *C. albicans*. *Candida* spp. establish a persistent lethal infection in *C. elegans*, as important components of *Candida* pathogenesis in mammals are also involved in nematode killing. In addition, *C. elegans* has proven useful for screening compounds for antifungal activity [21]. EA treatment at 4 μg/mL considerably protected *C. elegans* against *C. albicans* infection, prolonging larvae survival by 40% after 150 h postinfection. 

A literature survey revealed that although the in vitro bioactivity of the compounds has already been described, the antifungal activity of EA and CAPE against *C. auris* in a *G. mellonella* model has not been reported previously. Furthermore, to the best of our knowledge, this is the first study that investigated the anti-*C. albicans* properties of EA using *C. elegans*. Our findings corroborate with previous studies using different invertebrate models, since the protective effect of EA on a *Drosophila melanogaster* model and the efficacy of CAPE on *G. mellonella* and *C. elegans* models have already been described against *C. albicans* [21,33,85]. EA had a protective effect on *Drosophila melanogaster* from infection with *C. albicans* SC5314. Survival rates of 45%, 33%, and 34% were observed for the groups that received 3.2, 6.4, and 32 μg/mL of EA, respectively [33]. De Barros et al. (2021) found that the survival rate of *G. mellonella* larvae was 44.5% when treated with CAPE at 5 mg/kg, accompanied by a 2.07-fold increase in the number of hemocytes and stimulation of the expression of antifungal peptide genes galiomicin and gallerimycin, suggesting both antifungal and immunomodulatory activities [85]. We found that CAPE was not effective to protect *C. elegans* against *C. albicans*. However, another study reported that CAPE at 33 μg/mL was effective in the *C. elegans-C. albicans* DAY185 assay [21]. This difference can be related to different *C. albicans* strains which were used in the study. Research involving these invertebrate models demonstrate a remarkable correlation between in vitro susceptibility testing results and in vivo drug efficacy in invertebrate models. Overall, this research contributes to elucidate both the antifungal and antivirulence activity of CAPE and the antifungal effect of EA against drug-resistant *C. auris*.

## 5. Conclusions

In conclusion, ellagic acid and caffeic acid phenethyl ester showed antifungal activities against several *Candida* spp. tested through fungistatic and fungicidal effects, respectively. Both compounds may affect the fungal cell wall and thus warrant further investigations to clarify their specific role. CAPE was also able to reduce biofilm formation and both compounds decreased phospholipase production of *C. auris*. In addition, CAPE inhibited the virulence factors of *C. albicans*, such as yeast-to-hyphal transition and biofilm formation. Both compounds were not toxic to human erythrocytes. The in vivo analysis revealed the anti-infective potential of EA and CAPE by protecting *G. mellonella* from the pathogenicity of *C. auris* and *C. albicans*. In addition, the survival of *C. albicans*-infected *C. elegans* was prolonged after treatment with ellagic acid. Structural analogs can be synthesized to increase their efficacy and pharmacological properties, and future work is needed to evaluate their effects in mammalian models. Taking into account the lack of treatment options available for infections caused by multidrug-resistant *C. auris*, our findings indicate ellagic acid is a promising antifungal agent by inhibiting fungal growth, while CAPE inhibits virulence traits, including phospholipase secretion and fungal adhesion to surfaces.

## Figures and Tables

**Table 1:** Antifungal susceptibility of phenolic compounds against *Candida* spp.

Strains	MIC Values in μg/mL						
	Phenolic Compounds						
	CA	CAPE	CF	EA	EGCG	ENG	PD
*C. auris* CAU-01	4	1	128	0.125	1	>128	>128
*C. auris* CAU-02	64	8	>128	0.25	64	>128	>128
*C. auris* CAU-03	8	8	>128	0.25	16	>128	>128
*C. auris* CAU-04	64	16	>128	0.25	4	>128	>128
*C. auris* CAU-05	128	16	>128	0.25	32	>128	>128
*C. auris* CAU-06	128	32	>128	0.25	64	>128	>128
*C. auris* CAU-07	64	16	>128	0.25	32	>128	>128
*C. auris* CAU-08	128	16	>128	0.25	64	>128	>128
*C. auris* CAU-09	128	64	>128	0.25	64	>128	>128
*C. auris* CAU-10	128	64	>128	0.25	64	>128	>128
*C. albicans* CAL	>128	8	>128	0.5	16	>128	>128
*C. glabrata* CG	8	4	16	0.125	0.5	>128	>128
*C. krusei* CK	64	2	>128	0.125	2	>128	>128
*C. parapsilosis* CP	16	16	128	0.25	4	>128	>128
*C. tropicalis* CT	>128	32	>128	0.25	128	>128	>128

CA: caffeic acid, CAPE: caffeic acid phenethyl ester, CF: caftaric acid, EA: ellagic acid, EGCG: epigallocatechin gallate, ENG: engeletin, PD: polydatin.

**Table 2:** MIC values (μg/mL) of EA, CAPE and caspofungin against *C. auris* CAU-04 and *C. albicans* SC5314 and in the presence (+) and absence (−) of sorbitol (S).

Strain	MIC EA				MIC CAPE				MIC CPF (Control)			
	Day 2		Day 7		Day 2		Day 7		Day 2		Day 7	
	(−) S	(+) S	(−) S	(+) S	(−) S	(+) S	(−) S	(+) S	(−) S	(+) S	(−) S	(+) S
CAU	0.25	32	2	256	16	32	32	64	16	64	64	256
CAL	0.5	0.5	0.5	64	8	16	32	64	0.125	8	0.25	8

**Table 3:** MIC values (μg/mL) of EA, CAPE, and amphotericin B against *C. auris* CAU-04 and *C. albicans* SC5314 and in the presence (+) and absence (−) of ergosterol.

Strain	Drug	Ergosterol Concentration (μg/mL)					
		0	50	100	150	200	250
CAU	EA	0.25	0.25	0.25	0.25	0.25	0.25
	CAPE	16	16	16	16	16	16
	AMB (control)	0.5	4	8	16	32	64
	EA	0.5	0.5	0.5	0.5	0.5	0.5
CAL	CAPE	8	8	8	8	8	8
	AMB (control)	0.5	2	4	8	16	32

## Supplementary Materials

The following are available online at https://www.mdpi.com/article/10.3390/jof7090763/s1, Table S1: *Candida* strains used in this study. Table S2: Antifungal susceptibility of antifungal agents against *Candida* spp. Table S3: Anti-biofilm activities of EA, CAPE and FLC against *Candida* isolates. Figure S1: *C. albicans* filamentation assay. Figure S2: Toxicity of EA and CAPE and survival profile of *G. mellonella* when infected with *C. auris* and *C. albicans*.
