# Supplementary material for: Antifungal Activity of the Phenolic Compounds Ellagic Acid (EA) and Caffeic Acid Phenethyl Ester (CAPE) against Drug-Resistant Candida auris
Source: J Fungi (Basel). 2021 Sep 15;7(9):763. doi: 10.3390/jof7090763 (PMC8466507; doi:10.3390/jof7090763)
Supplement: Supplementary file 1 [file jof-07-00763-s001.zip › jof-1369202-supplementary.pdf]

Supplementary Materials

Table S1. *Candida* strains used in this study.

Strain	Description	Reference
CAU-01	AR-BANK#0381	CDC
CAU-02	AR-BANK#0382	CDC
CAU-03	AR-BANK#0383	CDC
CAU-04	AR-BANK#0384	CDC
CAU-05	AR-BANK#0385	CDC
CAU-06	AR-BANK#0386	CDC
CAU-07	AR-BANK#0387	CDC
CAU-08	AR-BANK#0388	CDC
CAU-09	AR-BANK#0389	CDC
CAU-10	AR-BANK#0390	CDC
CAL	WT	ATCC
CG	WT	ATCC
CK	WT	ATCC
CP	WT	ATCC
CT	WT	ATCC

CAU: *Candida auris*; CAL: *Candida albicans*; CG: *Candida glabrata;* CK: *Candida krusei*; CP: *Candida parapsilosis;* CT: *Candida tropicalis;* WT: Wild-type/

Table S2. Antifungal susceptibility of antifungal agents against *Candida* spp.

Strains	MIC values in μg/mL (interpretation)		
	Antifungals		
	**AMB**	**CPF**	**FLC**
CAU-01	0.5 (S)	0.125 (S)	4 (S)
CAU-02	0.5 (S)	0.5 (S)	16 (S)
CAU-03	0.5 (S)	0.25 (S)	128 (R)
CAU-04	0.5 (S)	16 (R)	128 (R)
CAU-05	0.5 (S)	0.5 (S)	>256 (R)
CAU-06	0.5 (S)	0.5 (S)	>256 (R)
CAU-07	1 (S)	0.5 (S)	8 (S)
CAU-08	1 (S)	0.25 (S)	>256 (R)
CAU-09	4 (R)	0.5 (S)	256 (R)
CAU-10	4 (R)	0.5 (S)	>256 (R)
CAL	0.5 (-)	0.125 (S)	0.25 (S)
CG	0.5 (-)	0.25 (I)	8 (SDD)
CK	0.5 (-)	0.5 (I)	32 (-)
CP	0.25 (-)	0.5 (S)	1 (S)
CT	0.25 (-)	0.25 (S)	64 (R)

Classification based on CDC and CLSI guidelines. S: sensitive. I: intermediate R: resistant. SDD: susceptible dose dependent. Breakpoints for *C. albicans*: CPF (S ≤ 0.25; R > 0.5) and FLC (S ≤ 2; R > 4); *C. glabrata*: CPF (S ≤ 0.125; R > 0.25) and FLC (S ≤ 32 SDD; R > 32); *C. krusei*: CPF (S ≤ 0.25; R > 0.5); *C. parapsilosis*: CPF and FLC (S ≤ 2; R > 4); *C. tropicalis*: CPF (S ≤ 0.25; R > 0.5) and FLC (S ≤ 2; R > 4); *C. auris*: AMB and CPF (S < 2; R ≥2), FLC (S < 32; R ≥ 32).
AMB: amphotericin B, CPF: caspofungin, FLC: fluconazole.

Table S3. Anti-biofilm activities of EA, CAPE and FLC against *Candida* isolates.

Strain	Agent	$MBIC_{50}$ (μg/mL)[1] determined by the use of		$MBEC_{50}$ (μg/mL)[2] determined by the use of	
		CV	XTT	CV	XTT
C. auris CAU-04	EA	>64	>64	>64	>64
	CAPE	64	32	>64	32

	Fluconazole	>64	>64	>64	>64
	EA	>64	>64	>64	>64
C. albicans SC5314	CAPE	32	8	>64	64
	Fluconazole	≤2	≤2	>64	>64

[1] $MBIC_{50}$: lowest concentration of the test compound that resulted in >50% inhibition of biofilm formation compared to the control,
[2] $MBEC_{50}$: The lowest concentration of the test compound resulted in >50% eradication of mature biofilm compared to the control.

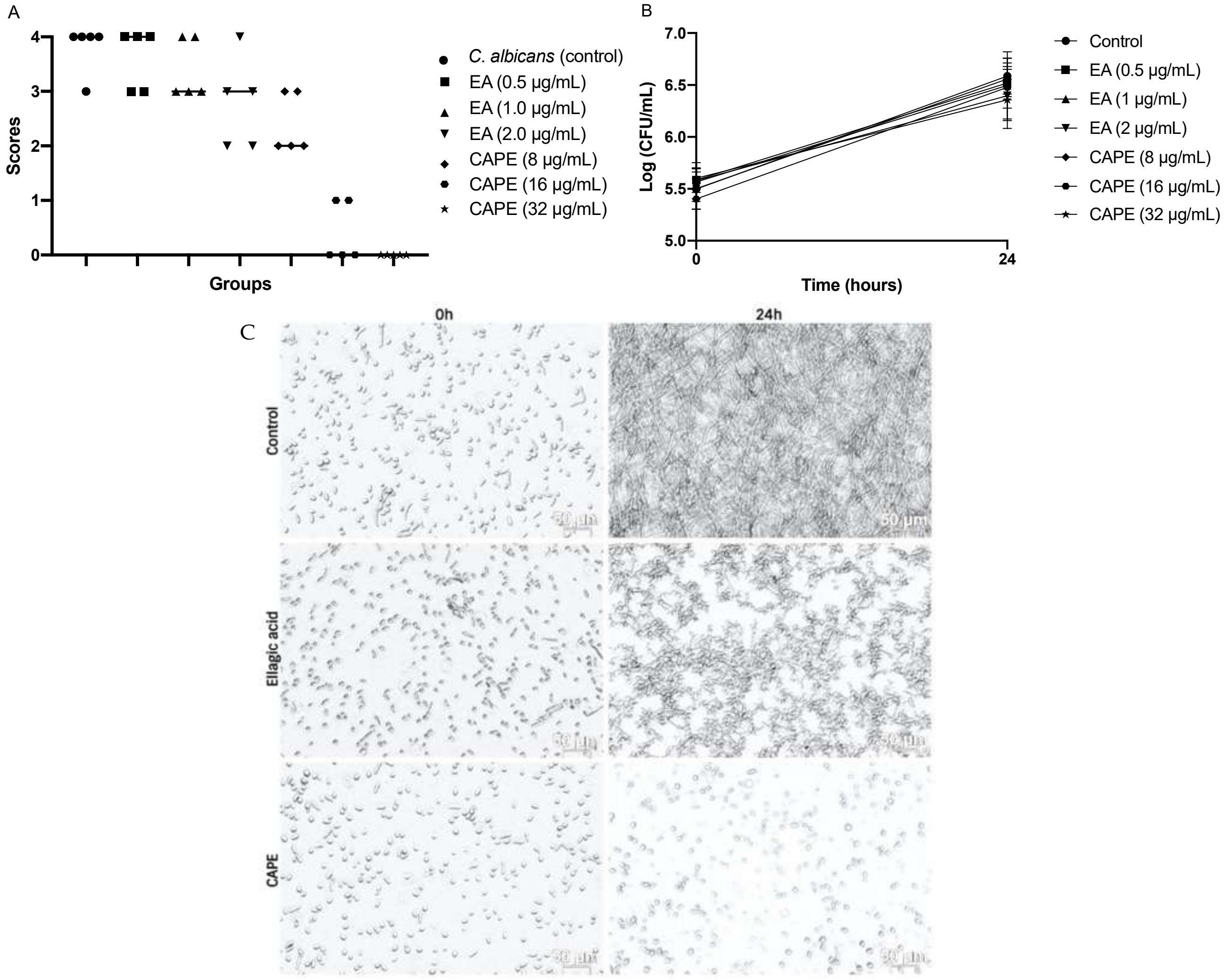

Figure S1. *C. albicans* filamentation assay. (A) Effects of EA and CAPE on *C. albicans* hyphal growth on liquid media. *C. albicans* was grown in RPMI-1640 medium in the presence of different concentrations of either EA or CAPE at 37°C for 24h. Median scores were obtained by hyphae counting of *C. albicans* alone (control group) and treatments (EA or CAPE). (B) Quantification of the CFU/mL (log) of *C. albicans* obtained in the non-treated control and experimental groups. No statistically significant differences were found. (C) Inhibitory effects of EA (4× MIC: 2 μg/mL) and CAPE (2× MIC: 16 μg/mL) on *C. albicans* filamentation. Aliquots were withdrawn and photographed using a bright field microscope.

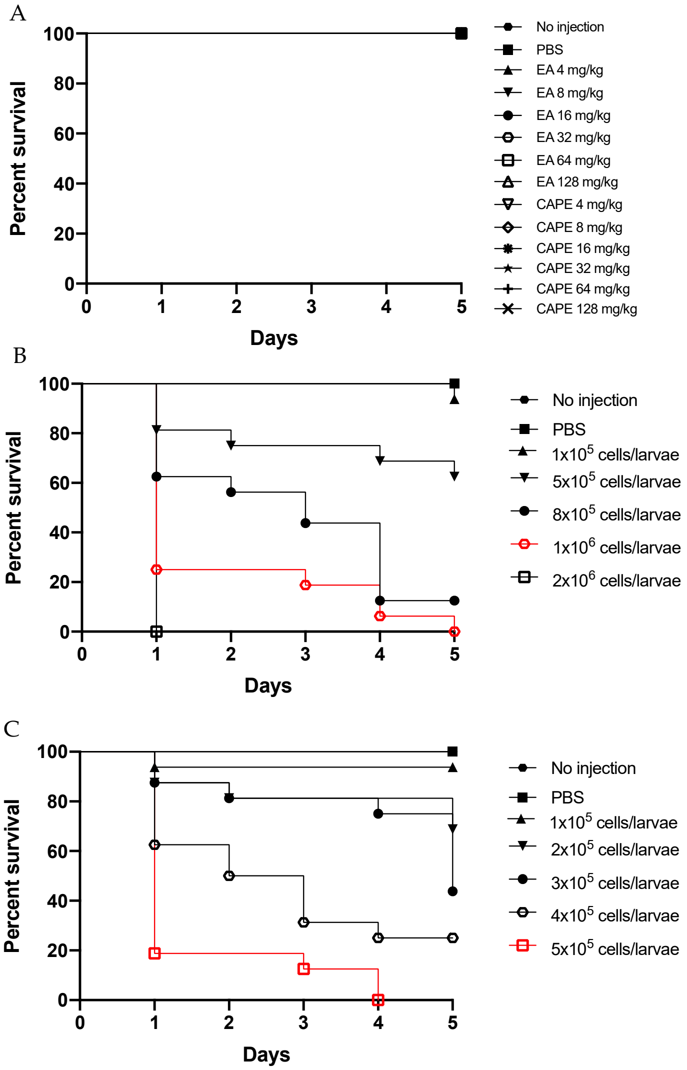

Figure S2. Toxicity of EA and CAPE and survival profile of *G. mellonella* when infected with *C. auris* and *C. albicans.* (A) Toxicity evaluation of EA and CAPE in *G. mellonella* model. No death was observed at the concentrations used. (B) Survival of *G. mellonella* larvae infected with different concentrations of *C. auris*. (C) Survival of *G. mellonella* larvae infected with different concentrations of *C. albicans*. The inoculum selected for further experiments are presented in red.

References

1. Alonso, G.C.; Pavarina, A.C.; Sousa, T. v.; Klein, M.I. A quest to find good primers for gene expression analysis of *Candida albicans* from clinical samples. *Journal of Microbiological Methods* **2018**, *147*, doi:10.1016/j.mimet.2018.02.010.
2. Clinical & Laboratory Standards Institute (CLSI) M27-A2: *Reference Method for Broth Dilution Antifungal Susceptibility Testing of Yeasts; Approved Standard - Second Edition. 1-51. 2002*; National Committee for Clinical Laboratory Standards, 2002; ISBN 1562384694.
3. Green, C.B.; Zhao, X.; Yeater, K.M.; Hoyer, L.L. Construction and Real-Time RT-PCR validation of *Candida albicans* PALS-GFP reporter strains and their use in flow cytometry analysis of *als* gene expression in budding and filamenting cells. *Microbiology* **2005**, *151*, doi:10.1099/mic.0.27696-0.
4. Li, D.-D.; Deng, L.; Hu, G.-H.; Zhao, L.-X.; Hu, D.-D.; Jiang, Y.-Y.; Wang, Y. Using *Galleria mellonella–Candida albicans* infection model to evaluate antifungal agents. *Biological and Pharmaceutical Bulletin* **2013**, *36*, doi:10.1248/bpb.b13-00270.

5. Liang, R.; Cao, Y.; Zhou, Y.; Xu, Y.; Gao, P.; Dai, B.; Yang, F.; Tang, H.; Jiang, Y. Transcriptional response of *Candida albicans* biofilms following exposure to 2-amino-nonyl-6-methoxyl-tetralin muriate. *Acta Pharmacologica Sinica* **2010**, *31*, doi:10.1038/aps.2010.33.
6. Nailis, H.; Kucharíková, S.; Řičicová, M.; van Dijck, P.; Deforce, D.; Nelis, H.; Coenye, T. Real-Time PCR expression profiling of genes encoding potential virulence factors in *Candida albicans* biofilms: identification of model-dependent and -independent gene expression. *BMC Microbiology* **2010**, *10*, doi:10.1186/1471-2180-10-114.
7. Rossoni, R.D.; Fuchs, B.B.; de Barros, P.P.; Velloso, M. dos S.; Jorge, A.O.C.; Junqueira, J.C.; Mylonakis, E. *Lactobacillus paracasei* modulates the immune system of *Galleria mellonella* and protects against *Candida albicans* infection. *PLOS ONE* **2017**, *12*, doi:10.1371/journal.pone.0173332.
